# Comparative Evaluation of Microhardness, Smear Layer Removal Efficacy and Depth of Penetration Using Punica granatum, Emblica officinalis and Sodium Hypochlorite As Endodontic Irrigants: An In Vitro Study

**DOI:** 10.7759/cureus.44760

**Published:** 2023-09-06

**Authors:** Deepika Lakshmaiah, Nancy Irudayaraj, Nivetha Ambeth, Anupama Ramachandran, Nikesh Sakthi, Nirmal Kumar

**Affiliations:** 1 Department of Conservative Dentistry and Endodontics, Chettinad Dental College and Research Institute, Chennai, IND; 2 Department of Conservative Dentistry and Endodontics, Ragas Dental College and Hospital, Chennai, IND

**Keywords:** emblica officinalis, smear layer, scanning electron microscope, punica granatum, microhardness, depth of penetration

## Abstract

Introduction

Root canal morphology tends to be complicated by nature and dealing with this intricacy can be challenging because it makes it difficult to completely disinfect the root canal space. The success of root canal therapy is also determined by the biomechanical preparation of the canal with the application of instruments and irrigating solutions. Due to the fact that the root dentin surface continues to interact with the irrigating solution during preparation, it's critical to evaluate the mechanical characteristics and smear layer removal. Though sodium hypochlorite (NaOCl) is the most commonly used irrigant due to its tissue-dissolving abilities, it has certain drawbacks which include the inability to remove the smear layer and also affects the mechanical properties of root dentin. To overcome these limitations, a variety of herbal substitutes like *Punica granatum* and *Emblica officinalis* which possess anti-bacterial and anti-fungal properties can be used as endodontic irrigants. Several studies on the anti-bacterial properties of natural irrigants of pomegranate and amla were reported. However, the mechanical properties and smear layer removal of *Punica granatum* and *Emblica officinalis* have not been explored in the field of endodontics.

Aim

The main aim of this in vitro study is to compare and evaluate microhardness, smear layer removal efficacy and depth of penetration of herbal and conventional irrigants.

Materials and methods

Thirty-six palatal roots of maxillary molars were decoronated and instrumented up to F3. These roots were sectioned longitudinally and divided into three test groups: Group 1: 12.5% *Punica granatum*; Group 2: 6.25% *Emblica* *officinalis*; control: Group 3: 2.5% NaOCl. All specimens were irrigated with 5ml of each irrigant for 5 minutes. Microhardness of root dentin was measured using a Vickers diamond intender, smear layer removal using a scanning electron microscope (SEM) and depth of penetration using a stereomicroscope. The data was analyzed using one-way ANOVA and the inter-group comparison using Tukey’s post hoc test.

Results

Statistical analysis was done using one-way analysis of variance (ANOVA) and Tukey’s post hoc test using SPSS software version 17.0 (SPSS Inc., Chicago ). The highest microhardness was seen in Group 1 (cervical: 53.8375 ± 1.35956, middle: 53.9875 ± 1.01761, apical: 53.6875 ± 1.63133) while Group 2 (cervical: 43.2750 ± 1.73596, middle: 43.3125 ± 1.17648, apical: 43.8000 ± ​​​​​​​1.32665) and Group 3 (cervical: 42.7250 ±​​​​​​​ 2.93391, middle: 41.9625 ±​​​​​​​ 1.66985, apical: 42.0250 ±​​​​​​​ 2.21085) showed significant reduction in root dentin hardness. Regarding* *smear layer removal Group 1 (1.3750 ±​​​​​​​ 0.51755), and Group 2 (1.2500 ±​​​​​​​ 0.46291) reveals greater smear removal in the middle third of the root dentin compared with Group 3 (2.3750 ±​​​​​​​ 0.51755), which showed the least smear layer removal. A greater depth of penetration was seen in Group 1 (0.5488 ± 0.05222) and Group 2 (0.5263 ±​​​​​​​ 0.05181) than in Group 3 (0.3087 ±​​​​​​​ 0.05743).

Conclusion

The present study reveals that the least reduction of microhardness was seen in *Punica granatum* followed by *Emblica officinalis* and NaOCl. The smear layer removal efficacy and depth of penetration were greater in* Punica granatum* and *Emblica officinalis* than in NaOCl. It was concluded that as these herbal irrigants are biocompatible agents, they can be considered for future use in root canal treatment.

## Introduction

The primary factor of endodontic failure is the retention of pathogens because of the complex structure of root canals [1]. An effective endodontic treatment requires the entire removal and disinfection of microorganisms and subsequent derivatives from the infected root canal, which is achieved in part by canal shaping but primarily through antimicrobial irrigants [2]. Root canals are prepared using manual and rotary instruments under copious irrigation. Regardless of the canal preparation procedure, 35% or more of root canal walls (along with the isthmus) were found to remain untouched in a study using micro-CT images taken before and after root canal shaping [3]. Thus, irrigation is an important component of endodontic treatment because it facilitates thorough disinfection beyond instrumentation.

Sodium hypochlorite (NaOCl) is a commonly used endodontic irrigant because of its antimicrobial and tissue-dissolving abilities [4]. Instrument corrosion, terrible taste, high toxicity [5], inability to remove the smear layer, loss of elastic modulus, and reduced flexural strength of dentin are some of the drawbacks of employing NaOCl [6]. As a result, many herbal alternatives have been explored as endodontic irrigants. The advantages of using herbal extracts in endodontics are that they have minimal cost, easy accessibility, extended shelf life, low toxic effects, lack of microbial resistance, are better tolerated by patients, and are eco-friendly [7].

*Punica granatum* (Pomegranate) exhibits antibacterial properties because of the presence of phytocompounds such as hydrolysable tannins, polyphenolics, and flavonoids [8,9]. The fruit of *Emblica officinalis* (Amla) contains antioxidants such as emblicanin A and B which are two hydrolyzable tannins that might explain Amla’s antibacterial properties [8-10]. Flavonoids and tannins cause increased antibacterial action against gram-positive anaerobes because of their ability to decrease a variety of microbial virulence processes, including suppression of biofilm formation, reduction of host ligand adherence, and neutralization of bacterial toxins [8,11,12]. The elimination of the smear layer from the root canal walls and dentinal tubules, as well as the reduction of harmful effects on dentin, is necessary for effective endodontic therapy [13,14].

As root dentin interacts with the irrigants during irrigation, it is important to assess the influence of irrigants on microhardness, smear layer removal efficacy and depth of penetration into dentin. This study investigates the effects of Punica granatum, Emblica officinalis, and NaOCl in dentinal root wall microhardness, smear layer removal efficacy, and depth of penetration. The null hypothesis was that there is no significant difference in microhardness, smear layer removal efficacy and depth of penetration of root dentin among the various irrigants.

## Materials and methods

The study received approval from the institutional human ethics committee (CARE IHEC-I/0363/21). Thirty-six permanent human maxillary first molars, freshly extracted for periodontal reasons, were used in this study. Maxillary molar teeth with straight roots extracted from patients between the age group of 25-50 years were selected. Carious teeth and teeth with developmental anomalies were excluded.

The palatal roots were decoronated at the cementoenamel junction using a diamond disc with a straight handpiece under constant water cooling. The root canal length was standardized to 14mm. A stainless steel K-type hand file #15 (Mani, Japan, 2022) was used to establish patency. Using the crown-down approach, the canals were prepared using Protaper Gold rotary NiTi files up to F3 (Dentsply Sirona, USA). During instrumentation, irrigation was performed using saline.

Preparation of herbal extracts

**Figure 1:**
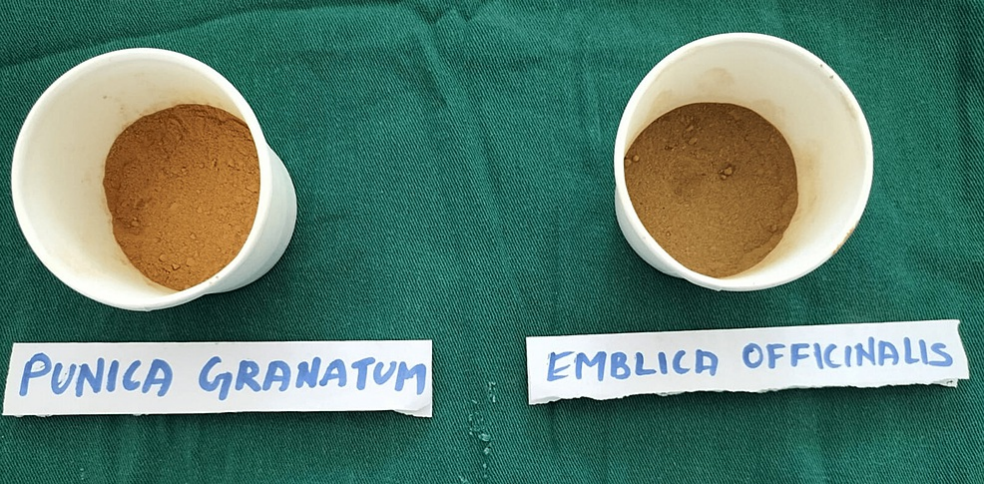
Prepared extract of Punica granatum and Emblica officinalis

*Punica* *granatum* (Pomegranate) peels and *Emblica* *officinalis* (Amla) fruits were sun-dried and ground into fine powder. The powders underwent cold maceration, which involved intermittent stirring with a sterile glass rod and being left undisturbed for 48 hours before being filtered through a sterile muslin cloth and heated in a water bath to 110°C to produce crude extracts. A total of 1.25g of dried amla crude extract is mixed with 20 ml of distilled water to obtain 6.25% of *Emblica officinalis *extract. And 2.5g of dried pomegranate crude extract is mixed with 20ml of distilled water to obtain 12.5% of *Punica granatum* extract (Figure 1) [8].

Sample distribution

The sample size was estimated using G* power software (version 3.1.9.2) (Erdfelder, Faul, & Buchner, 1996) and other input parameters:

1. Effect size - 0.55 (Payal et al., 2019)

2. Level of significance - 5%

3. Power (1-β) - 80%

The prepared specimens were divided randomly into three groups (n=12) based on the irrigant used: G1: 12.5% *Punica* *granatum,* G2: 6.25% *Emblica* *officinalis and* G3: 2.5% NaOCl.

Evaluation of microhardness

Pre-treatment Microhardness

Twelve prepared teeth were taken and longitudinal grooves were made on the buccal and lingual sides using a diamond disc without piercing the root canal space. Splitting of the teeth was done using a chisel into two halves. The specimens were stored in normal saline till use. Root segments were mounted using auto-polymerizing acrylic resin and further smoothened using fine emery papers. Microhardness values (M1) of all samples were measured with Vickers diamond intender at three different locations (cervical, middle and apical parts) using a 200g load and at 20s dwell time. The indentations were positioned 0.5 mm from the root canal surface at a depth of 100 µm from the pulp-dentin interface, with no overlap between them.

Post-treatment Microhardness

All these specimens were immersed in their respective irrigants for 5 minutes. Finally, they were rinsed with distilled water to avoid continuous exposure to the irrigants. Post-treatment microhardness values (M2) were recorded for each section after irrigation with corresponding irrigants. Microhardness values of all samples were measured similarly to the pretreatment microhardness. The decreased microhardness for each specimen was calculated.

Evaluation of smear layer removal

Twelve prepared teeth were irrigated with 5ml of respective irrigants for 5 minutes. Finally, rinsed with 1ml of distilled water for 1 minute. Splitting of teeth was done. Later, specimens were gold-platinum sputtered and then viewed under a scanning electron microscope (SEM). The middle third of the root canal was examined under 5000x magnification. The smear layer removal was measured by three blinded observers according to the criteria (Table 1) given by Torabinejad et al. [15].

**Table 1:** Evaluation criteria for smear layer removal

Scores	Evaluation Criteria
SCORE 1	No smear layer (no smear layer on the surface of the root canals; all tubules were clean and open)
SCORE 2	Moderate smear layer (no smear layer on the surface of root canal, but tubules contained debris)
SCORE 3	Heavy smear layer (the smear layer covered the root canal surface and tubules)

Evaluation of depth of penetration

The crystal violet dye was made to flow from the apical end of each prepared tooth (12 teeth) for about five seconds. The teeth were then soaked in the dye for two days and washed for 10 minutes under tap water. The teeth were then irrigated with 5ml of respective irrigants for 5 minutes. Splitting of the teeth was done as mentioned previously. Each sample was then evaluated in the middle third of the canal using a Stereomicroscope (Leica Microsystems, Germany) under 20x magnification. ImageJ software (National Institutes of Health, USA) was used to calculate the penetration depth. The depth of the bleaching zone was calculated in millimetres.

Statistical analysis

The results were collected and tabulated. Mean and standard deviation were calculated for all the groups. Statistical analysis was done using analysis of variance (one-way ANOVA) and Tuckey’s post hoc test. Statistical significance was considered at a level of P<0.05. Data were analyzed using Statistical Package for the Social Sciences (IBM SPSS Statistics for Windows, IBM Corp., Version 17.0, Chicago).

## Results

Microhardness

The variations between the M1 and M2 scores at all three levels of root dentin were examined using one-way ANOVA. All the irrigating solutions used resulted in reduced microhardness compared with baseline values. They showed a statistically significant difference (*p*<0.05) between the initial and post-treatment microhardness values. In Table 2 Group 2 (cervical: 43.2750 ± 1.73596, middle: 43.3125 ± 1.17648, apical: 43.8000 ± 1.32665) and Group 3 (cervical: 42.7250 ± 2.93391, middle: 41.9625 ± 1.66985, apical: 42.0250 ± 2.21085) showed reduced microhardness compared with Group 1 (cervical: 53.8375 ± 1.35956, middle: 53.9875 ± 1.01761, apical: 53.6875 ±​​​​​​​ 1.63133). The post-treatment microhardness values were most decreased NaOCl and *Emblica officinalis *whereas, *Punica granatum* showed the least changes.

**Table 2:** Inter-group comparison for pre- and post-microhardness using ANOVA Statistically significant p<0.05; SD: standard deviation, ANOVA: analysis of variance

Groups			Mean	Standard Deviation	Std. Error Mean	P-value
Group 1	Cervical	Pre	56.6500	0.77275	0.27321	0.001
		Post	53.8375	1.35956	0.48068	
	Middle	Pre	57.4125	1.31740	0.46577	0.001
		Post	53.9875	1.01761	0.35978	
	Apical	Pre	57.1750	0.89562	0.31665	0.001
		Post	53.6875	1.63133	0.57676	
Group 2	Cervical	Pre	55.4250	1.54712	0.54699	0.001
		Post	43.2750	1.73596	0.61376	
	Middle	Pre	56.2875	1.70749	0.60369	0.001
		Post	43.3125	1.17648	0.41595	
	Apical	Pre	55.2375	1.67156	0.59099	0.001
		Post	43.8000	1.32665	0.46904	
Group 3	Cervical	Pre	53.0250	2.52516	0.89278	0.001
		Post	42.7250	2.93391	1.03730	
	Middle	Pre	52.9500	0.85021	0.30059	0.001
		Post	41.9625	1.66985	0.59038	
	Apical	Pre	52.9375	0.97825	0.34586	0.001
		Post	42.0250	2.21085	0.78165	

Smear layer removal

**Figure 2:**
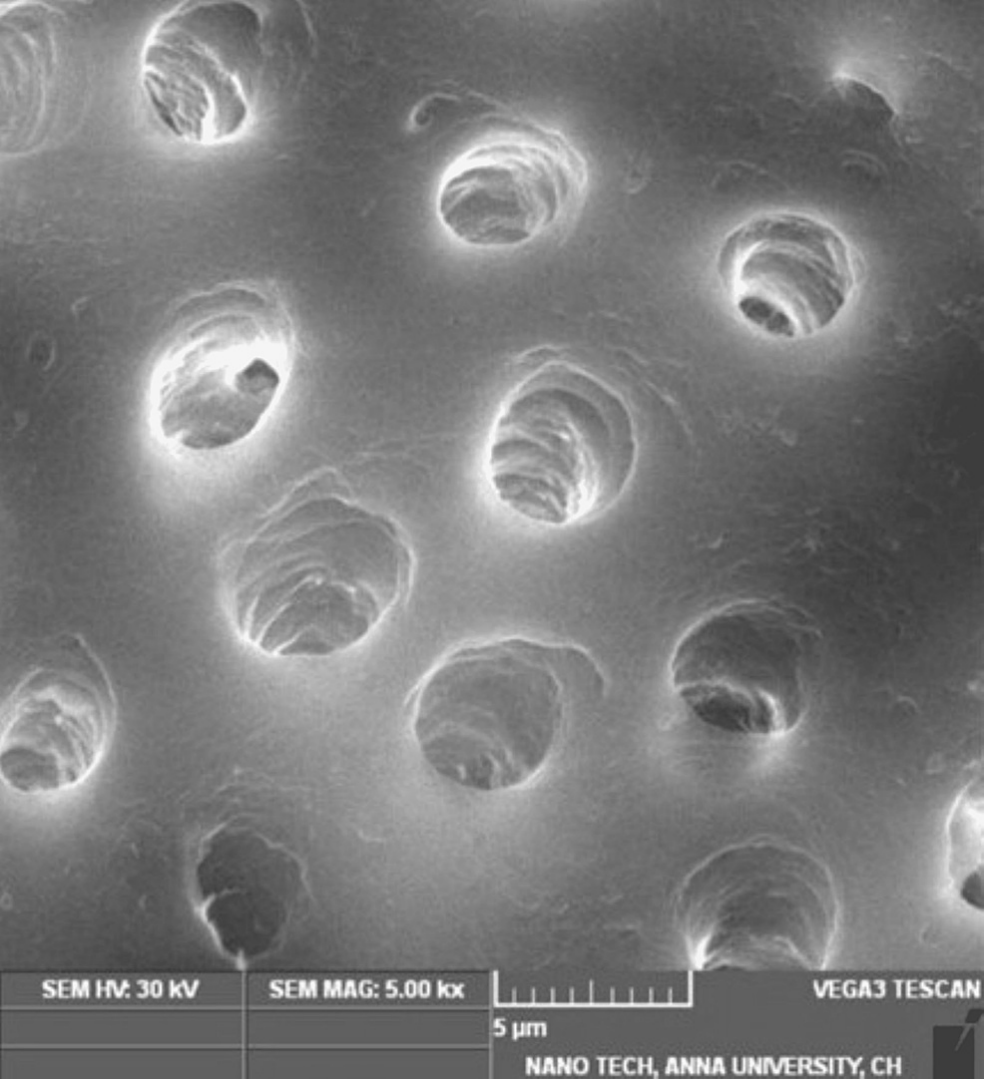
SEM photomicrograph example of root canal wall treated with 12.5% Punica granatum in 5000x with score 1 showing patent orifices of dentinal tubules and no smear layer particles in the examined field. SEM: scanning electron microscope

**Figure 3:**
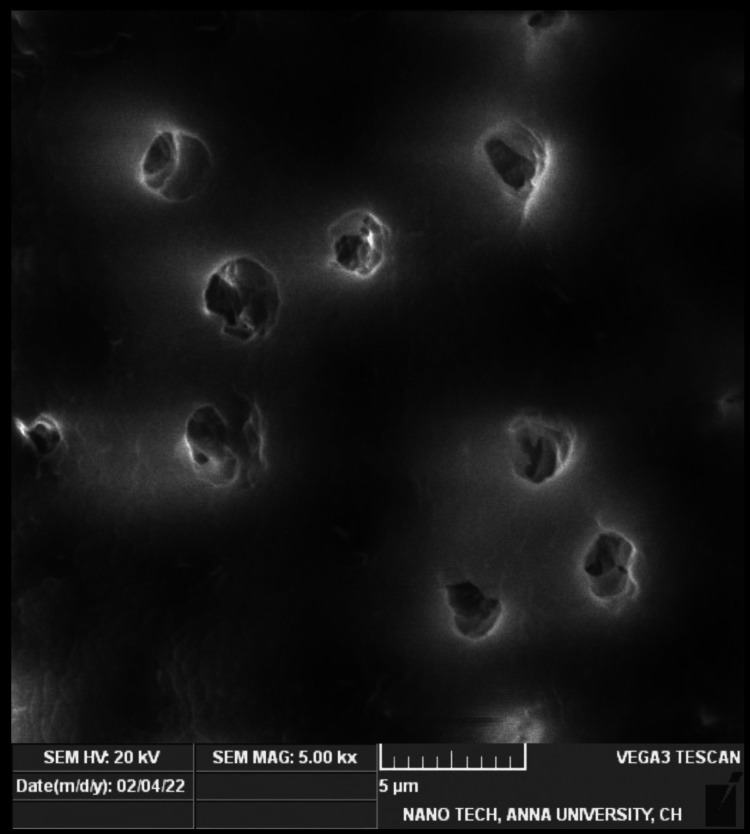
SEM photomicrograph of an example of root canal wall treated with 6.25% of Emblica officinalis in 5000x with score 1 showing patent orifices of dentinal tubules and no smear particles in the examination field. SEM: scanning electron microscope

**Figure 4:**
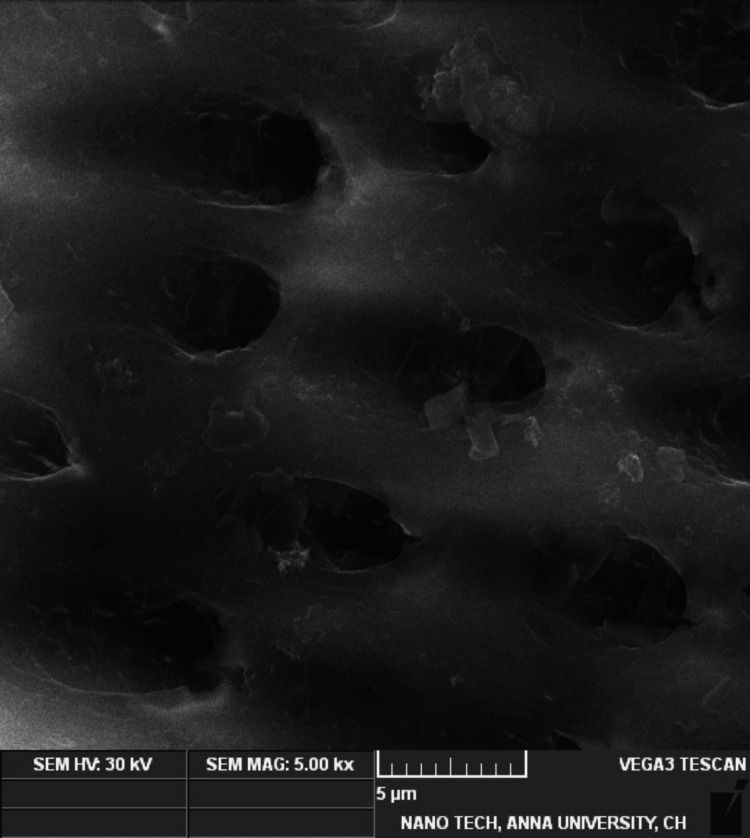
SEM photomicrograph example of root canal wall treated with NaOCl in 5000x with score 3 showing patchy distribution of smear layer up to 50% of root canal area. SEM: scanning electron microscope, NaOCL: sodium hypochlorite

The mean value for the smear layer removal of all three groups was analyzed at 5000x magnification. Table 3 depicts the mean values of Group 1 (1.3750 ± 0.51755), and Group 2 (1.2500 ± 0.46291) revealing greater smear removal in the middle third of the root dentin compared with Group 3 (2.3750 ±​​​​​​​ 0.51755), which showed the least smear layer removal. Group 1 (Figure 2) and Group 2 (Figure 3) showed that the orifices of the dentinal tubules were patent and no smear layer was present (score of 1 with no smear layer present). The NaOCl group (Figure 4) depicts a variable distribution of smear particles over the dentinal tubules (score of 3 with up to 50% of root canal area). However, using one-way ANOVA and Tukey's post hoc test, Groups 1 and 2 showed better smear layer removal, followed by Group 3, which showed the least removal.

**Table 3:** Inter-group comparison for smear layer removal Statistically significant p<0.05; F: ANOVA (analysis of variance) value

Groups	Mean	Standard Deviation	95% Confidence Interval for Mean		F	P-value
			Lower Bound	Upper Bound		
Group 1	1.3750	0.51755	0.9423	1.8077	12.16	0.001
Group 2	1.2500	0.46291	0.8630	1.6370		
Group 3	2.3750	0.51755	1.9423	2.8077		

Depth of penetration

**Figure 5:**
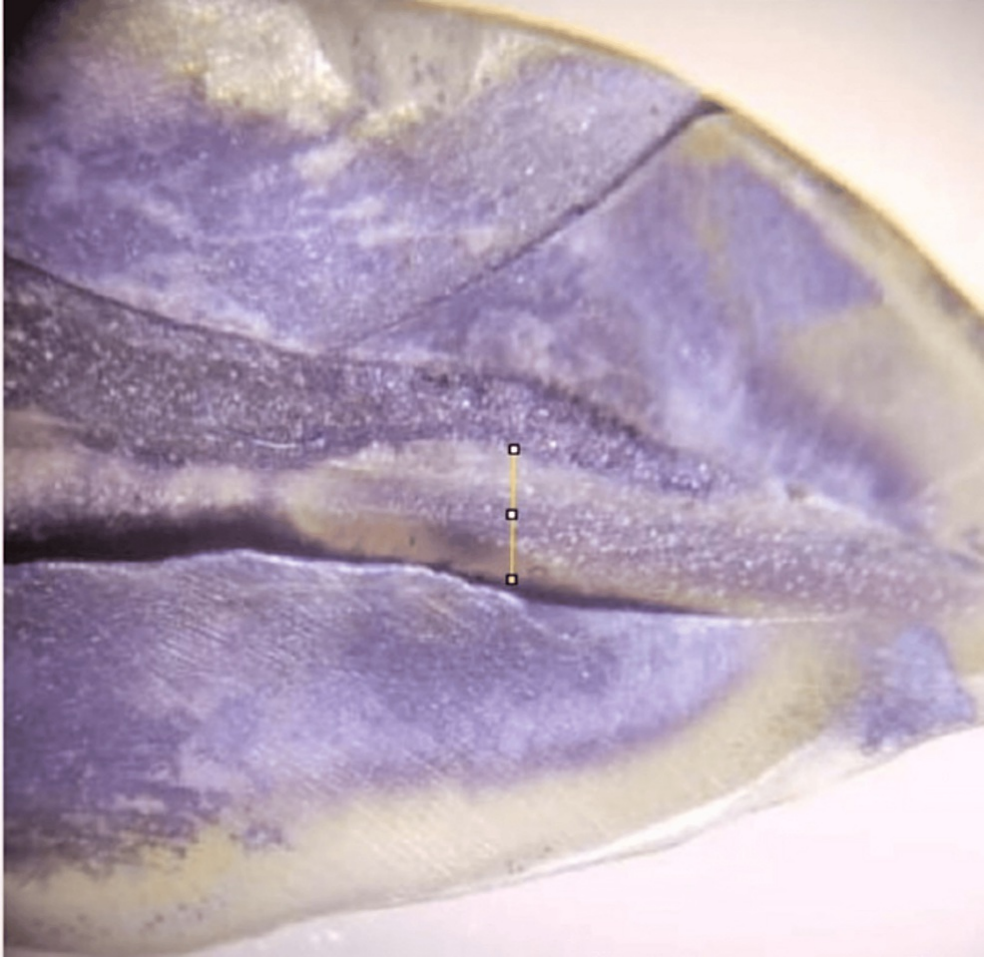
Example of depth of penetration determined under Stereomicroscope

Data for depth of penetration were calculated in millimetres (Figure 5). ImageJ software was used to calculate the depth of penetration at the middle third of the canal. The values were statistically analyzed using ANOVA and tabulated in Table 4. The mean value of Group 1 (0.5488 ± 0.05222) and Group 2 (0.5263 ±​​​​​​​ 0.05181) showed greater depth of penetration than Group 3 (0.3087 ± 0.05743), which was statistically significant (P<0.05). There was no difference between Groups 1 and 2.

**Table 4:** Inter-group comparison for smear layer removal Statistically significant p<0.05; SD: standard deviation; F: ANOVA (analysis of variance) value

Groups	Mean	SD	95% Confidence Interval for Mean		F	P-value
			Lower Bound	Upper Bound		
Group 1	0.5488	0.05222	0.5051	0.5924	48.41	0.0001
Group 2	0.5263	0.05181	0.4829	0.5696		
Group 3	0.3087	0.05743	0.2607	0.3568		

## Discussion

The complex root canal system must be cleaned and shaped properly for effective endodontic therapy. Root canal bacteria play a key role in the development of dental pulp inflammation and necrosis, resulting in periapical lesions [15,16]. *Enterococcus faecalis *is one of the most resistant strains in the canal and causes re-infection. [17]. Eradication of microorganisms during endodontic treatment is essential and depends on biomechanical preparation, irrigation, and intra-canal medications [18]. An ideal irrigant should have antibacterial properties, and the potential to remove the leftover necrotic pulpal tissue and cause only mild irritation to the periapical tissues. Existing formulations mostly comprise antibiotics, antibacterial agents, surfactants, and alcohol, which have been shown to be cytotoxic and only partially effective in eliminating oral infections. Herbal medicines are increasingly becoming the treatment of choice as endodontic irrigants [19].

In vitro experiments have shown tannins containing herbal extracts are substances that have a variety of antibacterial and antifungal properties. *Punica granatum *and *Emblica officinalis* both contain a significant amount of tannins and other components [7,8]. Payal et al. [8] compared the antibacterial efficacy of 6.25% *Emblica* *officinalis*, 12.5% *Punica* *granatum*, and 1% NaOCl against *E*. *faecalis* and found that these aqueous extracts have good antimicrobial properties as endodontic irrigants.

NaOCl is the most commonly used irrigant because of its greater tissue-dissolving ability [4]. However, it has a number of drawbacks, including the ability to irritate the tissue and produce dentin collagen denaturation, significant cell toxicity, and disintegration of the stem cells at the periapex, causing an unpleasant taste and perhaps causing allergies and leading to hypochlorite mishaps [20]. Currently, herbal compounds are preferred as endodontic irrigants. Hence, the present study evaluated the effect of various endodontic irrigating solutions such as 12.5% *Punica* *granatum*, 6.25% *Emblica* *officinalis*, and 2.5% NaOCl on microhardness, smear layer removal, and depth of penetration of irrigants. In addition to its advantages, irrigants may have negative effects on root dentin [21].

Irrigating solutions can affect the microhardness of root dentin, which in turn impacts the clinical performance of teeth that have had endodontic treatment, according to Saha SG et al. [22]. Even though reduced microhardness makes instrumentation easier, it might weaken the root structure [22]. Dentin is made up of 22% organic material, primarily collagen type I. Its depletion produces morphologic disarray and mechanical property changes [23,24]. According to Pashley et al. [25], tubular density impacts microhardness, as the density of the tubules increases, dentin microhardness decreases, likely because of a reduction in intertubular dentin and an increase in individual tubular diameter [25]. NaOCl causes a reduction in the organic content of root dentin. It also has the propensity to degrade the long peptide chains and chlorinate the protein terminal groups, which results in further breakdown of N-chloramines into other species. According to Dayal C et al. [26], instrumentation and irrigation with NaOCl alter the dentin’s biomechanical properties. In our study, NaOCl lowered the microhardness values. *Emblica* *officinalis* showed a decrease in microhardness which can be related to its pH value of 2.5 (acidic pH) [10]. This results in alterations in the ratio of the organic to the inorganic component of radicular dentin, which in turn affects the microhardness. *Punica* *granatum* showed a slightly acidic pH (3.25-4.14), which may be the reason for a decreased tendency to reduce microhardness and can be considered an effective endodontic irrigant [27].

The removal of the smear layer from the root canal walls and dentinal tubules is another crucial part of effective endodontic treatment. Because it prevents a hermetic closure between the guttapercha and the sealer, the smear layer must be removed [28]. The results of the present study showed a greater smear layer in *Emblica* *officinalis* and *Punica* *granatum* than in NaOCl. The pH of Amla is acidic because of the presence of gallic acid. The availability of calcium ions from hydroxyapatite for chelation decreases as the pH increases. As a result, a higher dissociation of the acidic irrigant results in greater attraction of calcium ions [7,10]. *Punica* *granatum* also exhibits greater smear layer removal because of its slightly acidic pH. The presence of ascorbic acid and flavonoids in these herbal irrigants may be the reason for their efficient removal of the smear layer [28].

An increased penetration depth of irrigants into dentinal tubules may increase the success rate of endodontic therapy. The shaping procedure showed inadequate debridement and regions unaffected by the manual K-files or rotary instruments, elimination of bacteria is mostly influenced by the depth to which irrigants penetrate to scavenge microorganisms that are firmly embedded inside the dentinal tubules [29]. According to Bystrom and Sundqvist [30], the diffusion of anti-microbial agents such as intracanal irrigants and sealers in the dentinal tubules can be initiated because of the presence of the smear layer. The dentinal tubules were stained with crystal violet dye because it allows for greater stereomicroscopic visualization [30]. The herbal irrigants showed better depth of penetration into the dentinal tubules than NaOCl. Greater penetration of irrigants into the dentinal tubules may be because of the efficient removal of the smear layer. The results of this study show that *Punica* *granatum* and *Emblica* *officinalis* can be used as alternative irrigants. The main benefits of utilizing herbal substitutes are their accessibility, affordability, longer shelf life, low toxicity, and absence of known microbial resistance.

Limitations

This study was in vitro the outcomes in actual patients may differ. Hence, lengthy in vivo investigations must be conducted to assess their effectiveness.

Another drawback of the study is that the irrigants were used individually rather than in combination. Hence, further investigations must be conducted to examine the interactions between these natural extracts and various other irrigants.

To assess their toxicity on live cells, toxicological studies must be conducted on living organisms.

## Conclusions

Herbal irrigants like *Punica* *granatum* and *Emblica* *officinalis* have proved to be advantageous as endodontic irrigants. The present study reveals that the least reduction of microhardness was seen in *Punica* *granatum* followed by *Emblica* *officinalis* and NaOCl. The smear layer removal efficacy and depth of penetration were greater in *Punica* *granatum* and *Emblica* *officinalis* than in NaOCl. It was concluded that these herbal irrigants are biocompatible agents, it can be considered for future use in root canal treatment.

## References

[1] Siqueira JF Jr, Rôças IN (2008). Clinical implications and microbiology of bacterial persistence after treatment procedures. J Endod.

[2] Mohammadi Z, Soltani MK, Shalavi S (2014). An update on the management of endodontic biofilms using root canal irrigants and medicaments. Iran Endod J.

[3] Peters OA, Schönenberger K, Laib A (2001). Effects of four Ni-Ti preparation techniques on root canal geometry assessed by micro computed tomography. Int Endod J.

[4] Zehnder M (2006). Root canal irrigants. J Endod.

[5] Mohammadi Z (2008). Sodium hypochlorite in endodontics: an update review. Int Dent J.

[6] Sim TP, Knowles JC, Ng YL, Shelton J, Gulabivala K (2001). Effect of sodium hypochlorite on mechanical properties of dentine and tooth surface strain. Int Endod J.

[7] Prabhakar J, Senthilkumar M, Priya MS, Mahalakshmi K, Sehgal PK, Sukumaran VG (2010). Evaluation of antimicrobial efficacy of herbal alternatives (Triphala and green tea polyphenols), MTAD, and 5% sodium hypochlorite against Enterococcus faecalis biofilm formed on tooth substrate: an in vitro study. J Endod.

[8] Jain PA, Tejaswi S, Parinitha MS, Shetty S, Ambikathanaya UK (2019). Comparative evaluation of antibacterial activity of Punica granatum, Acacia nilotica and Emblica officinalis against Enterococcus faecalis and their smear layer removal ability when used as endodontic irrigants: an in-vitro study. Int J Res Rev.

[9] Aravindraj S, Preethi M, Sivapathasundharam B (2017). Antimicrobial effects of Punica granatum Extracts on Staphylococcus aureus, Streptococcus mutans, Lactobacillus acidophilus, Enterococcus faecalis and Candida albicans. Int J Curr Microbiol App Sci.

[10] Karan YB, Shalini A, Tanaya K, Shobha B (2015). Comparative evaluation of the efficacy of three anti-oxidants vs NaOCl and EDTA: used for root canal irrigation in smear layer removal-sem study. Int J Pharm Pharm Sci.

[11] Cushnie TP, Lamb AJ (2005). Antimicrobial activity of flavonoids. Int J Antimicrob Agents.

[12] Daglia M (2012). Polyphenols as antimicrobial agents. Curr Opin Biotechnol.

[13] Chhabra N, Gyanani H, Kamatagi L (2015). Smear layer removal efficacy of combination of herbal extracts in two different ratios either alone or supplemented with sonic agitation: an in vitro scanning electron microscope study. J Conserv Dent.

[14] Sebatni MA, Kumar AA (2017). Smear layer removal efficacy of herbal extracts used as endodontic irrigants: an in vitro study. Endodontol.

[15] Torabinejad M, Khademi AA, Babagoli J (2003). A new solution for the removal of the smear layer. J Endod.

[16] Chow AT, Quah SY, Bergenholtz G, Lim KC, Yu VS, Tan KS (2019). Bacterial species associated with persistent apical periodontitis exert differential effects on osteogenic differentiation. Int Endod J.

[17] Pinheiro ET, Gomes BP, Ferraz CC, Sousa EL, Teixeira FB, Souza-Filho FJ (2003). Microorganisms from canals of root-filled teeth with periapical lesions. Int Endod J.

[18] Nangia D, Nawal RR, Talwar S (2020). Evaluation of apical extrusion and cone-beam computed tomography assessment of irrigant penetration in oval-shaped canals, using XP Endo Finisher and EndoActivator. J Conserv Dent.

[19] Flemingson Flemingson, Emmadi P, Ambalavanan N, Ramakrishnan T, Vijayalakshmi R (2008). Effect of three commercial mouth rinses on cultured human gingival fibroblast: an in vitro study. Indian J Dent Res.

[20] Mohmmed SA, Vianna ME, Penny MR, Hilton ST, Knowles JC (2017). The effect of sodium hypochlorite concentration and irrigation needle extension on biofilm removal from a simulated root canal model. Aust Endod J.

[21] Philip PM, Sindhu J, Poornima M, Naveen DN, Nirupama DN, Nainan MT (2021). Effects of conventional and herbal irrigants on microhardness and flexural strength of root canal dentin: an in vitro study. J Conserv Dent.

[22] Saha SG, Sharma V, Bharadwaj A (2017). Effectiveness of various endodontic irrigants on the micro-hardness of the root canal dentin: an in vitro study. J Clin Diagn Res.

[23] Bertassoni LE (2017). Dentin on the nanoscale: hierarchical organization, mechanical behavior and bioinspired engineering. Dent Mater.

[24] Moreira DM, Almeida JF, Ferraz CC, Gomes BP, Line SR, Zaia AA (2009). Structural analysis of bovine root dentin after use of different endodontics auxiliary chemical substances. J Endod.

[25] Pashley D, Okabe A, Parham P (1985). The relationship between dentin microhardness and tubule density. Endod Dent Traumatol.

[26] Saripella MKR, Konagala RK, Anupreeta A, Varma UL, Ramesh P, Shaik J (2020). Evaluation of root dentin microhardness prepared with hand and two rotary file systems with 2.5% sodium hypochlorite irrigation solution - an in vitro study. IP Ann Prosthodont Restor Dent.

[27] Akbarpour V, Hemmati K, Sharifani M (2009). Physical and chemical properties of pomegranate (Punica granatum L.) fruit in maturation stage. Am Euras J Agric Environ Sci.

[28] Shahravan A, Haghdoost AA, Adl A, Rahimi H, Shadifar F (2007). Effect of smear layer on sealing ability of canal obturation: a systematic review and meta-analysis. J Endod.

[29] Lussi A, Nussbächer U, Grosrey J (1993). A novel noninstrumented technique for cleansing the root canal system. J Endod.

[30] Bystrom A, Sundqvist G (1985). The antibacterial action of sodium hypochlorite and EDTA in 60 cases of endodontic therapy. Int Endod J.

